# Gum Chewing, Added to Conventional Feeding, Reduces Risk of Post-Operative Ileus after Elective Hip and Knee Arthroplasty Procedures in Elderly Population: A Protocol for a Parallel Design, Open-Label, Randomized Controlled Trial

**DOI:** 10.29337/ijsp.158

**Published:** 2021-08-09

**Authors:** Obada Hasan, Laraib Mazhar, Ahsun Jiwani, Dilshad Begum, Riaz Lakdawala, Shahryar Noordin

**Affiliations:** 1 Fellow Orthopaedic Oncology, University of Iowa, PK; 2 Research Associate, Department of Medicine, Aga Khan University Karachi, PK; 3 Junior Epidemiologist, The Indus Hospital Research Centre Karachi, PK; 4 Manager & Senior Instructor, Clinical Trials Unit, Aga Khan University Karachi, PK; 5 Associate Professor, Department of Surgery, Medical College, Aga Khan University Karachi, PK; 6 Associate Professor & Service Line Chief, Department of Surgery, Medical College, Aga Khan University Karachi, PK

**Keywords:** Bowel movement, arthroplasty, gum chewing, gut mobility, length of hospital stay, gut motility

## Abstract

**Introduction:**

Postoperative ileus (poi) is defined as a temporary cessation of bowel movement after a surgical procedure. Cessation of bowel movement not only leads to disturbing constipation but also may lead to nausea, loss of appetite, and food intolerance. Literature reports “sham feeding” (gum-chewing) effect as an increase in chewing and saliva which enhances the gastric emptying and overall motility of gut as a cephalic phase of digestion. Therefore, we aim to assess the effect of adding gum-chewing to the conventional postoperative feeding regimen on restoring postoperative bowel function and length of stay in hospital of patients undergoing elective hip arthroplasty.

**Methods and analysis:**

This is a single-center, open-label, parallel design, superiority randomized-controlled trial with 2 treatment arms. The primary and secondary outcomes will be the time interval in hours from the end of surgery until the passage of flatus and the time interval in hours from the end of surgery until the passage of stool. Statistical analysis will be done using STATA software. Length of stay will be calculated by Kaplan–Meier analysis, with unadjusted comparison of groups by Mantel–Cox log rank test. Risk ratios for the time-to-become ileus free and time-to-discharge from hospital will be calculated by Cox regression modeling. P value as 0.05 or less will be taken as significant.

**Ethics And Dissemination:**

This protocol is exempted from Ethical review at this stage however all the required approvals will be taken from the ethical review committee before starting the study. Informed consent will be taken form the patient to enroll him/her in the study. Results of the study will be disseminated to the study participants, public health, and clinical professionals. The results would also be published in a reputable international journal.

**Trial Registration:**

This trial is registered on *clinicaltrials.gov* with ID: NCT04489875.

**Highlights:**

- Cessation of bowel movement not only leads to disturbing constipation but also may lead to nausea, loss of appetite, and food intolerance.
- These patients tend to have more pain scores and dissatisfaction with the surgical management and team.
- The “sham feeding” (gum-chewing) effect causes an increase in chewing and saliva formation and therefore enhances the gastric emptying and overall motility of gut as a cephalic phase of digestion even in non-gastro or colorectal surgeries.
- We hypothesize that there is an association between gum chewing and the relief from postoperative ileus in hip arthroplasty patients.

## 1. Introduction

Traditionally, post-operative ileus(poi) is defined as temporary cessation of bowel movement after surgical procedure [1]. Surgical procedures include not only abdominal or colorectal procedures but non abdominal procedures as well, like cesarean sections, arthroplasty, and urological and thoracic procedures [1,2,4]. It is a self-limiting condition for most of patients. It is an under-estimated problem which may affect patient condition directly or indirectly [3]. Cessation of bowel movement does not lead only to a disturbing constipation, but also may lead to nausea, loss of appetite and food intolerance. These patients tend to have more pain scores and dissatisfaction with the surgical management and team [5]. At the moment, no protocol is present to prevent this annoying short-term complication. mechanism behind this condition is caused by decrease in vagal parasympathetic stimulation [3,6]. Here comes the “sham feeding” (gum-chewing) effect where increase chewing and saliva enhance the gastric emptying and overall motility of gut as a cephalic phase of digestion even in non-gastro or colorectal surgeries [2,7,8]. This effect is studied thoroughly in gastric, colorectal, and gynecological procedures. There is scarcity about its effect following orthopedic procedures specifically hip and knee arthroplasty. We therefore hypothesize that sham feeding by using gum chewing will enhance patient’s gut motility, reduce the time to first flatus and stool, enhance the food tolerance and reduce length of hospital stay.

### 1.1 Rationale

With increasing pressure on limited health resources and continually needing to improve the quality of patients’ preoperative experience, interventions with the potential to limit the discomfort of postoperative stay are gaining popularity. The rationale of this study is to accelerate patient recovery and reduce his discomfort and length of hospital stay, which will further reduce the financial burden, by potentially cheap and simple remedy.

### 1.2 Study objectives

Primary objective is to assess the effectiveness of adding gum-chewing to the conventional postoperative feeding regimen on restoring postoperative bowel function in patients undergoing elective hip and knee arthroplasty. Secondary objective is to assess the effect of gum-chewing on length of hospital stay in patients undergoing elective hip arthroplasty.

## 2. Methods

### 2.1 Study design

This study will be a parallel, open-label, superiority randomized-controlled trial at a single hospital. The two arms will be equally allocated on a 1:1 ratio into intervention and control groups.

### 2.2 Interventions and Duration

Behavioral intervention type (Sugarless Gum-chewing) starting the morning after surgery when the patient is fully awake and allowed to start taking feed orally (which usually starts within 6–10 hours after surgery). Patient will be given the gum to chew for 15min minimum duration each time, 3 times/day before the usual time of meal, until the first flatus/passing stool (whatever happen first) in addition to conventional postoperative feeding schedule. Control group will have conventional feeding schedule without added gum. Secondary outcomes will be the postoperative interval until the first passage of stool in hours as well as postoperative hospital stay in days (Surgery to discharge).

### 2.3 Primary and secondary Outcome Measures

Primary outcome will be the time interval in hours from end of surgery till passage of flatus, which is reported subjectively by the patient. Patients will be instructed to make note of the time when flatus is passed first time after surgery. Secondary outcomes will be the postoperative interval until the first passage of stool in hours as well as postoperative hospital stay in days (Surgery to discharge).

### 2.4 Sample size calculations

To the best of author’s knowledge, this is the first research to study the effect of gum-chewing post-arthroplasty procedure on time to flatus. Literature reported numerous studies on same objective but post abdominal and colorectal surgeries which gave minimum mean time to flatus post-surgery of 67 hours. In arthroplasty no handling of abdominal viscera is there, and we assume time of bowel return to function is considerably less. Hence, considering the rarity of previous literature and the number of cases to be operated in 6 months, investigators decided to recruit and study all eligible patients during the study period from July 2021 to December 2021. Expected number in each arm will be of 50 patients.

### 2.5 Selection and enrollment of subjects

#### 2.5.1 Inclusion Criteria

Adult patients aged between 50 to 70 years old undergoing elective primary hip and knee joint replacement surgery with ASA grade I–III and under general anesthetic with/without neuraxial anesthesia will be included.

#### 2.5.2 Exclusion Criteria

Refusal of consent established naso-gastric/gastrostomy feeding, or unsafe swallow due to any neurological disease will be excluded. Investigators will exclude patients with bowel disease other than peptic ulcer, history of chronic constipation more than 3 days before surgery, inability to chew gum due to dental issues, traumatic and revision cases of arthroplasty, adjuvant surgical procedures (Abdominal, thoracic, etc.) beside the primary arthroplasty procedure.

### 2.6 Study Enrollment Procedures

Enrollment will be a continuous process with screening and enrolling eligible patients admitted through clinic electively for primary THR and TKR at the Aga Khan University. Consent will be taken in clinic along with the consent for the procedure by the primary surgeon or his senior resident as per the department policy. Participants will be explained in detail about the procedure as well as the research purpose and intervention detail and duration. We will try to make sure that we purchase the gums from the same vendor for all participants. This study will be funded by the Department of surgery at the Aga Khan University.

### 2.7 Handling of Study Interventions

Sugarless gum will be purchased in bulk after confirming the expiry date and will be kept at the Clinical Trial Unit (CTU) team, dispatching the required gums as per patients’ needs and request from the ward nurse, preferably one at a time. As the intervention is behavioral, it will not be suitable to blind/mask the study intervention.

### 2.8 Adherence Assessment

Compliance of the participants in intervention group will be assessed during normal routine ward rounds at morning and evening. As this is a superiority trial, this adherence along with the dropouts will be incorporated in the analysis phase using with the intention to treat principle as it is the most conservative approach in this scenario and the least to be biased. The patient will be encouraged to keep a diary, otherwise the gum will be provided by the nursing team. As per the hospital policy, nursing team provides all oral medications and help the patients to take them, same procedure will be followed for the gums.

### 2.9 Pre-Randomization Screening

Screening will involve the routine history taking and physical examination by the doctor and nursing staff in the clinic. Screening will not involve performing procedures that are not part of routine patients’ management. Special attention will be given to the dental history and whether patient is able to chew gum or not.

### 2.10 Randomization

After screening and eligibility, patients will be randomly allocated by 1:1 ratio to the Group A gum-chewing (Gum) or Group B control (No gum) groups using a computer-generated randomization sequence by the CTU at the Aga Khan University, which they will provide to the nurse/PI by telephone after patients’ admission in hospital. Patients will be followed from the time they reach the ward after surgery till their hospital discharge, which is usually 7 days.

### 2.11 Intervention Discontinuation Evaluations

We defined “intervention discontinuation” as those participants who do the gum-chewing less than the required time (minimum 15 min) for 3 consecutive timings for any reason. They will be encouraged to resume study intervention and if they refuse then they will be treated as per protocol during analysis phase.

### 2.12 Management of adverse experiences

No published side effects of this intervention are known or anticipated. In case of any un-anticipated events, PI, CTU and ERC will be informed via telephonic call and written report will be submitted afterwards via email. Interim analysis will be shared with the appointed Data and Safety Monitoring Board (DSMB).

### 2.13 Statistical considerations

Categorical data will be summarized as absolute values (percentage). Continuous data will be presented as mean+/– SD if normally distributed and median +/– IQR if not. Length of stay will be calculated by Kaplan–Meier analysis, with unadjusted comparison of groups by Mantel–Cox log rank test. Risk ratios for the time-to-become ileus free and time-to-discharge from hospital will be calculated by Cox regression modeling, considering the following independent variables: age, gender, operation type, diabetes mellitus, preoperative cardiovascular disease (ischemia/heart failure/dysrhythmias), patient-controlled analgesia (PCA) opiate use and presence/absence of chewing gum. P value as 0.05 or less will be taken as significant. Statistical analysis will be done by STATA software version 15.

### 2.14 Data collection, site monitoring, and adverse experience reporting

Standardized questionnaire and training of data collectors to be done by the PI to ensure reliability and validity of the study. Data collection will be done by the data collectors under supervision of the PI and CTU. Questionnaires will be checked for consistency and logical entries. Data entry will be done, and counter checked by the PI on regular intervals. Data collected will be kept confidential without identifiable information of patients who are identified by a number assigned. The hard copy forms will be retained in a secured location with the PI after data entry into computer software and will be kept as per hospital protocol.

### 2.15 Data Management

#### 2.15.1 Data Editing

Data editing will be done both by the data collector or PI at the time of data collection. The questionnaire will be checked for consistency, completeness, and logical entries. To obtain smooth data entry, PI will be carrying out another data editing after completion of data collection process. Data editing is a continuous process, at the completeness of form and then at the time of data entry and at end of data entry.

#### 2.15.2 Data Entry

Data will be entered twice. First entry will be done by the data entry operator and second will be done by PI. The data entry operator will be trained for data entry in terms of reading the filled data forms and its code book. The operator will be asked to enter data for 30 study participants under the supervision of the PI and the mistakes will be communicated. Both the entries will be checked by PI for consistency and errors will be corrected by referring to the forms.

#### 2.15.3 Data cleaning

Data will be cleaned by checking missing values, double and un-realistic entries.

#### 2.15.4 Data storage

Data collected will be kept confidential without identifiable information of patients who are identified by a number assigned. The hard copy forms will be retained in a secured location with lock and key mechanism with the PI after data entry into computer which also secured by username and password. The data will be available for AKU Ethical Review Committee on request and might be published in journal without disclosing any identifiable information of patients. Questionnaires will be stored for 5 years after the study is completed as per policy of the institute.

#### 2.15.5 Quality Assurance

Quality of information gathered will be given priority and subsequently its integrity will be maintained. To ensure data quality, revisions and formatting of the instruments will be carried out to reduce error. PI will do the random checks for the completeness and consistency of data collection forms or request one of the study committee members to that. The data collection forms will be reviewed on daily basis by principal investigator for the purpose of good quality of data collection. Mistakes identified in this manner will be assessed immediately and measures will be taken to correct them. Training will be given to the data collectors to ensure that there are no problems with the quality of data collection.

#### 2.15.6 Study Modification/Discontinuation

The study can be modified or discontinued at any time by the CTU, ERC or the DSMB as part of their duties to ensure that research subjects are protected.

## 3. Ethical Considerations

This protocol is exempted from Ethical review at this stage however all the required approvals will be taken from the ethical review committee before starting the study.

### 3.1 Informed Consent

Informed consent will be taken from the patient as per routine protocol before the arthroplasty procedures in the hospital. Research objectives, methodology, risks, and benefits will be explained in detail. Consent will only be taken by the primary investigator at his clinic or the surgical resident on call after patient’s admission in hospital, as per routine care.

### 3.2 Subject Confidentiality

All laboratory specimens, evaluation forms, reports, video recordings, and other records that leave the site will be identified only by the Study Identification Number (SID) to maintain subject confidentiality. All records will be kept in a locked file cabinet. All computer entry and networking programs will be done using SIDs only. Clinical information will not be released without written permission of the subject, except as necessary for monitoring by IRB, the FDA, the NINDS, the OHRP, the sponsor, or the sponsor’s designee.

## 4. Dissemination of research findings

Publication of the results of this trial will be governed by the policies and procedures developed by the Executive Committee. Any presentation, abstract, or manuscript will be made available for review by the ERC and CTU prior to submission.

## Additional File

The additional file for this article can be found as follows:

10.29337/ijsp.158.s1 CONSORT Checklist. The additional file includes the CONSORT checklist of reporting randomized trials.
